# Portrayal of oral hygiene and risk behaviours in animated movies

**DOI:** 10.3389/froh.2023.1116717

**Published:** 2023-07-05

**Authors:** George Kitsaras, Michaela Goodwin

**Affiliations:** Dental Health Unit, Division of Dentistry, The University of Manchester, Manchester, United Kingdom

**Keywords:** oral hygiene behaviours, risk behaviours, sugar, children, animated movies

## Abstract

**Background:**

Behaviours depicted in movies and TV shows can significantly affect one's behaviour. Children are particularly susceptible to these effects as their habits are still forming. Oral hygiene behaviours play a crucial role in preventing or slowing down the progression of dental diseases, which are among the most common yet preventable diseases in the world. Therefore, it is important to understand if popular movies include oral hygiene behaviours or risk-related behaviours, which can in effect influence children's behaviour.

**Aim:**

The aim of this study is to review the top grossing animated movies of all time to record and collect on screen portrayals of oral hygiene practices and risk behaviours related to oral health.

**Methodology:**

Top 30 highest grossing animated feature films (over 40 min duration) were coded using a structured coding instrument to capture oral hygiene and risk-related behaviours related to oral health. Two coders performed coding using the standardised instrument.

**Results:**

Overall, 93% of behaviours were coded as a risk behaviour, with 7% coded as positive oral hygiene behaviour. Within the risk behaviour category, the majority (74%) were based around the consumption of sugar with risk behaviour occurring in 23 out of 30 movies (76%), while oral hygiene practices occurred in 6 out of 30 movies (20%); one movie depicted neither oral hygiene nor oral health risk behaviours. About 53% of behaviours were purely visual, 10% verbal, and 37% a combination of verbal and visual. Anthropomorphic characters and movie settings resulted in more behaviours related to oral health, either hygiene or mainly risk behaviours, depicted.

**Conclusion:**

Despite their importance in shaping habits and attitudes, animated movies portrayed a significant number of risk behaviours related to oral health with depiction of beneficial behaviours remaining limited. Consideration should be given on how to best portray behaviours that promote and enhance optimal oral hygiene behaviours to achieve and sustain better oral health for children.

## Introduction

Movies and cinema have been one of the greatest developments of the last century. For decades, movies have reflected societal changes, promoted critical thinking, and showcased some of the best and the worst parts of humanity (1). Movies as well as other forms of media, through their wide reach, beyond demographic, economic, and other differences have a crucial role in today's world in reflecting ongoing developments and issues as well as promoting values and shaping behaviours (2).

Previous research has highlighted the potential role movies, cinema, and television can play in forming and shaping behaviours. Most importantly, past research has also shown the importance these media can have in forming and shaping health-related behaviours (3). From alcohol intake to tobacco use, smoking and food intake television and movies have been found to play a crucial role increasing the adoption of unhealthy behaviours in both adults and children (3–6).

Movies and television can affect one's behaviours, including health-related behaviours through different mechanisms predominately to norm setting, habit formation, and imitation. Additionally, behavioural theories such as the social learning theory can provide further explanation on the effect movies and television can have one one's behaviours. Learning new or shaping existing behaviours can be influenced by vicarious observations in socially mediated contexts (7, 8). Through these observations, one can view actions and their consequences, if actions of unhealthy behaviours are not followed by direct consequences, then potentially unhealthy behaviours will not be mitigated (7, 8). On the other hand, the absence of beneficial behaviours severely limits learning through observations due to the lack of available prompts and cues (7, 8). Observation learning and its effect on behaviours has been closely studied within commercials (2, 4–6, 9, 10) and TV shows (11, 12) with a particular focus on alcohol use, smoking, and other risky behaviours.

Nevertheless, evidence is scarce regarding unhealthy behaviours in animated movies that are particularly focused on children and younger audiences (13–15). Children are a sponge ready to absorb information from various sources; this information in return helps children to increase their understanding of the world around them, helps them to conceptualise values and principles for life, as well as promote their understanding on how to behave in various scenarios (13–15). Younger children are much more likely to absorb and relate to the information provided to them while habits and behaviours still forming (13–15). Studies have shown a strong effect of commercials on children who were found to identify and learn from animated characters they observe (16). Movies, contrary to advertisements, can have an even greater impact on children as they deploy richer and more engaging techniques, last for a longer period, incorporate plots, and have an altogether stronger persuasive power.

Through the range of health-related behaviours children learn from an early age, oral hygiene is a key one. Dental diseases represent one of the most common yet mostly preventable diseases in the world with over half a billion children worldwide experiencing dental decay (17). The effects of dental diseases like dental decay can be detrimental to children, their wellbeing, and their development (18–21). Dental diseases can cause pain, sleepless nights, affect their school performance, create agitation, and impact their behaviour as well as their self-confidence (18–21). When left untreated, dental decay can progress and result in unnecessary extractions. For example, in England, in 1 year, over 60,000 children will have teeth extracted under general anaesthetic in hospital resulting in pain, anxiety, hospital stays, and unnecessary disruption to children's lives as well as costing the public finances millions of pounds in healthcare costs (22).

To prevent dental decay and dental diseases and to slow down the progression of disease, optimal oral hygiene practices are vital. These include brushing teeth twice a day using a fluoride toothpaste, flossing, and using mouthwash (23). Additionally, limiting sugar intake is crucial in maintaining good oral hygiene and avoiding disease (24). As dental disease is preventable through optimal behaviours, it is therefore essential to consider if and how such behaviours are portrayed in animated movies, which are geared mostly towards children and younger audiences. To this date, no study has evaluated the presence (or absence) of oral hygiene practices and risk-related oral health behaviours such as sugar intake, alcohol, and smoking in animated movies to establish what children and younger audiences might be viewing.

An opportunity therefore exists to review existing, high grossing animated movies, establish their role in communicating oral hygiene practices and oral health risk behaviours, and creating a dialogue on the responsibility of movies towards oral health. This work can lead to future developments where, for example, set criteria for portraying oral hygiene practices and risk behaviours are developed to create consistency on the messages that younger audiences receive.

## Aim

The aim of this study is to review the top grossing animated movies of all time to record and collect on screen portrayals of oral hygiene practices and risk behaviours related to oral health.

## Methodology

### Sample

Top 30 highest grossing animated movies of all time as indicated through IMDb (Internet Movie Database, the most-visited and trusted film, television series, podcast, and streaming content database in the world) and other relevant and reliable sources. Only animated movies (i.e., feature films) are considered for this work; a feature film needs to have a running time of at least 40 min. Table 1 presents an overview of the movies used in the study. Despite rich offerings on TV and online shows, for this study, we prioritised animated feature-length films given the wide reach of movies across physical cinemas, online platforms, and other forms of content sharing of movie clips and scenes (i.e., YouTube, TikTok, etc.).

**Table 1:** List of movies used in the study.

ID	Name of movie	Year of release
1	Frozen II	2019
2	Frozen	2013
3	Incredibles 2	2018
4	Minions	2015
5	Toy Story 4	2019
6	Toy Story 3	2010
7	Despicable Me 3	2017
8	Finding Dory	2016
9	Zootopia	2016
10	The Lion King	1994
11	Despicable Me 2	2013
12	Finding Nemo	2003
13	Shrek 2	2004
14	Ice Age: Dawn of the Dinosaurs	2009
15	The Secret Life of Pets	2016
16	Ice Age: Continental Drift	2012
17	Inside Out	2015
18	Shrek the Third	2007
19	Coco	2017
20	Shrek Forever After	2010
21	Madagascar 3: Europe's Most Wanted	2012
22	Monsters University	2013
23	Ne Zha Zhi Mo Tong Jiang Shi	2019
24	Up	2009
25	Kung Fu Panda 2	2011
26	Ice Age: The Meltdown	2006
27	Big Hero 6	2014
28	Moana	2016
29	Kung Fu Panda	2008
30	The Incredibles	2004

### Data collection

For this study, the unit of analysis are all scenes where oral hygiene practices and/or risk behaviours are displayed. A scene is defined as a section in the movie where there are not any changes in the continuity of the action or any breaks in the interaction between characters caused by other characters or events. Behaviours need to be actively present during a scene in dialogue (verbal presentation), action (visual presentation), or a combination of both. If a background character is displaying a behaviour, then this will not be counted. Supplementary material A provides a copy of the coding instrument used in the study.

For each exposure (i.e., scene), four key variables will be coded using the coding instrument presented below:

(a) Nature of behaviour: oral hygiene behaviour and/or risk behaviour (sugar consumption, smoking, alcohol).
(b) Mode of presentation: verbal, visual (action), or a combination of both.
(c) Valence of behaviour: positive, negative, or neutral (how other characters react to the displayed behaviour).
(d) Nature of character: good or evil.
(e) Anthropomorphic nature (human like or not human like).

### Data analysis

One movie was not included in the analysis as it had no oral hygiene or oral health risk behaviours present. Descriptive statistics (raw data and frequency) are presented. The authors conducted a simple bivariate analysis by cross tabulating the measure of interest (nature of behaviour) against other recorded variables including nature of character, whether characters were considered anthropomorphic, and valance of behaviour. All statistical analyses were performed using SPSS version 27.

## Results

### Oral hygiene vs. risk-related behaviours

The study found that when looking at behaviours related to oral health, 93% were coded as a risk behaviour, with 7% coded as positive oral hygiene behaviour. Within the risk behaviour category, the majority (74%) were based around the consumption of sugar (Table 2). Risk behaviour occurred in 23 out of 30 movies (76%), while oral hygiene practices occurred in 6 out of 30 movies (20%); 1 movie depicted neither oral hygiene nor oral health risk behaviours. About 53% of behaviours were purely visual, 10% verbal, and 37% a combination of verbal and visual.

**Table 2:** Descriptive statistics of the nature of the behaviour.

Oral hygiene practices		
Practice	*N*	%
Brushing	5	5
Flossing	1	1
Use sippy cup with water	1	1
Total oral hygiene practices	7	7
Risk behaviours		
Behaviour	*N*	%
Sugar intake (total)	74	74
Eating sugar	64	
Drinking sugary drinks	6	
Both eating and drinking	2	
Other form of sugar intake	2	
Smoking	2	2
Alcohol	16	16
Other	1	1
Total risk behaviours	93	93

To understand if the behaviours were associated with particularly character attributes within the movie, associations between nature of the character and the behaviour were explored (see Table 3).

**Table 3:** Nature of character by nature of behaviour.

	Good	Evil	Total %
Oral hygiene practice	4 (57.1%)	3 (42.9%)	7
Risk behaviour	75 (81.5%)	17 (18.5%)	92
	79	20	99

X^2^ (1) = 2.398, *p* = 0.121.

No evidence of an association between whether a character was good or evil and if they performed a positive oral hygiene practice or negative oral health risk behaviours was observed. Therefore, the “good” characters carried out many of both risk behaviours and oral hygiene practices. In fact, the good characters carried out over 80% of the risk behaviours coded.

Characters within the movie were also divided into whether they were considered anthropomorphic (human in nature) or not. This explored against the nature of behaviour divided into positive oral hygiene practices or oral health risk behaviours (Table 4).

**Table 4:** Anthropomorphic nature of character by nature of behaviour.

	Human like	Not human like	Total %
Oral hygiene practice	4 (57.1%)	3 (42.9%)	7
Risk behaviour	80 (86.0%)	13 (14.0%)	93
	84	16	100

X^2^ (1) = 4.040, *p* = 0.044.

A significant association was observed between the anthropomorphic nature of characters and the “negative” risk behaviour or “positive” oral hygiene practice carried out. About 86% of risk behaviours were carried out by human like characters compared to 14% of characters who were not human like. A more even distribution was observed between the oral hygiene practices, but the overall proportion (7%) was low compared to the proportion of risk behaviours observed within the movies.

Finally, the valance of behaviour (whether it could be considered positive, negative, or neutral) was explored against the nature of behaviour (Table 5).

**Table 5:** Valance of behaviour by nature of behaviour.

	Positive	Negative	Neutral	Total %
Oral hygiene practice	2 (28.6%)	0	5 (71.4%)	7
Risk behaviour	20 (21.5%)	29 (31.2%)	44 (47.3%)	93
	22	29	49	100

X^2^ (2) = 3.103, *p* = 0.212.

No evidence of an association between whether a character was good or evil and if they performed a positive oral hygiene practice or negative oral health risk behaviours were observed. While all oral hygiene practice was coded as either positive or negative, 31% of risk behaviours were portrayed as negative with 69% of risk behaviours were portrayed as positive or neutral.

## Discussion

This study explored the oral health-related behaviours portrayed in the 30 highest grossing children's animated movies. The results revealed that oral health risk behaviours appeared in over three-quarters of the 30 movies viewed. The main risk factor was the consumption of food containing sugar. This occurred in all different characters whether they were considered “good” or “evil” with the valance of risk behaviours depicted as both positive and negative.

The depiction of risk-related behaviours can potentially have an important, negative effect on shaping children's habits leading to potentially higher consumption of sugar and engagement in other negative behaviours regarding their oral health. Diet, especially sugar intake, has a long-established link to dental diseases especially dental caries (24, 25). Free sugars, i.e., natural sugars that have been broken down as well as added sugars in food and drinks, have a damaging effect on dental health due to their high cariogenic load (26). The amount of free sugars consumed as well as the frequency of free sugar consumptions are both important factors in disease manifestation and progression; higher amount consumed and more frequent consumption can lead to higher incidence of disease (24).

Unsurprisingly, characters that were depicted as more human like, which included those who were human or had anthropomorphic characteristic, such as animals acting as humans (Zootropolis) or characters such as the ogre Shrek, carried out more oral health-related behaviours. There was an association when comparing the oral health-related behaviour (risk or oral hygiene) by the anthropomorphic nature of the group with a much higher proportion of risk behaviours seen in the group that were deemed to be more human like.

These results provide an additional insight into the exposure children have, in relation to either positive or negative oral health behaviours. This is an important concept as children learn through observing and imitating behaviour (7). Social learning theory has described how the characteristics of a model's behaviour can have a significant influence over the observers learning. This has been described in relation to gender or sex, social power, or racial identity (27) and, therefore, characters who are like humans or portray more human like characteristics may also have a greater influence in observers imitating behaviour. The modelling of behaviour through favourite characters or characters that are perceived as “good” could be stronger as children identify more with positive characters. If these characters perform behaviours that are portrayed positively (even if their actions could be considered as risks such as the consumption of sugar), they may be more likely to perform them in real life (7, 8).

This study found that oral health-related behaviours were more likely to be depicted visually with just 10% involving only a verbal component. As children are more likely to recognise and remember audio-visual information compared to audio alone, it can be observed that within the movies included, both the positive oral hygiene and negative risk behaviours could have an increased chance of being perceived and recollected by those watching (28).

Reflecting on the detrimental role of free sugars and diet more broadly on dental disease, a range of campaigns are underway to help limit the amount of sugar intake and promote overall nutrition in children (29). In light of the findings from this study, animated movies still have a long way to go in order to better reflect key priorities for child wellbeing and development as particularly reflected on the need to limit sugar intake and the consequences of sugar consumption. Programs and movies for children need to provide an improved balance between positive and negative oral health behaviours and a clearer association of the negative impact of risk behaviours.

### Strengths and Limitations

The main strength of this study is the deployment of a detailed coding instrument that allowed multiple types of target behaviours to be captured effectively including verbal, visual, and combined portrayals across selected films. Limitations of this study include (a) the focus on animated movies vs. other forms such as TV shows, internet shows, etc.; (b) the focus on only the top 30 highest grossing films of all time leaving out some very popular movies from the analyses; and (c) the use of English-language films with no consideration of other languages.

### Future directions

In the future, this work can be expanded with the inclusion of more animated movies as well as TV shows focusing on children. We believe that there might be a different standard of information portrayed in TV shows targeting younger children, for example, CBBC and CBeebies shows in the United Kingdom might have further considerations on how to showcase the importance of oral hygiene practices and risk behaviours related to oral health. Additionally, expanding the review to the portrayal of dental professionals within animated movies and whether they are portrayed as an evil or good character (on a personal and not purely professional manner) could provide further insights. Also, introducing child- and parent-focused surveys to understand what impact (if any) these portrayals have on their attitudes and behaviours could strength future research in this area.

This review, with its focus on top grossing animated movies, has found very little incidence of beneficial oral hygiene behaviours portrayed. Additionally, in future work, consensus building can take place on how to showcase oral hygiene behaviours (and risk behaviours) in movies and shows. This is the first, small step in better understanding the potential effect of animated movies on a vital oral health-related behaviour.

## Conclusion

Movies, television, online shows, and media have recently and gradually become a constant factor in children's lives. Given their power to shape habits, immense and widespread reach across platforms, demographics, and cultures, it is essential to understand and highlight the need for better practices, habits, and behaviours to be showcased and reflected in movies and shows. This study explored the portrayal of oral hygiene and risk behaviours related to oral health in highest grossing animated movies. The findings clearly indicate that on one hand, oral hygiene behaviours such as brushing teeth remain at best very limited, while on the other hand, risk behaviours related to oral health, especially sugar intake, remain very common. More research is needed to better map and understand the wider landscape outside of movies. Steps to address how such behaviours are portrayed in movies need to follow.

## Data Availability

The raw data supporting the conclusions of this article will be made available by the authors, without undue reservation.

## References

[1] Carroll N. The power of movies. Daedalus. (1985) 114(4):79–103. 20025008.

[2] Johnson BC. Introduction: movies as edutainment. Counterpoints. (2015) 474:1–10. 45178401.

[3] Anderson PM Butcher KF. Childhood obesity: trends and potential causes. Future Child. (2006) 16(1):19–45. 10.1353/foc.2006.0001 16532657

[4] Dalton MA Beach ML Adachi-Mejia AM Longacre MR Matzkin AL Sargent JD Early exposure to movie smoking predicts established smoking by older teens and young adults. Pediatrics. (2009) 123(4):e551–8. 10.1542/peds.2008-2102 19336346 PMC2758519

[5] Engels RCME Hermans R van Baaren RB Hollenstein T Bot SM. Alcohol portrayal on television affects actual drinking behaviour. Alcohol Alcohol. (2009) 44(3):244–9. 10.1093/alcalc/agp003 19237443

[6] van Hoof JJ de Jong MDT Fennis BM Gosselt JF. There's alcohol in my soap: portrayal and effects of alcohol use in a popular television series. Health Educ Res. (2009) 24(3):421–9. 10.1093/her/cyn037 18640968

[7] Bandura A. Observational learning. In: The international encyclopedia of communication [Internet]. John Wiley & Sons, Ltd (2008). Available at: https://onlinelibrary.wiley.com/doi/abs/10.1002/9781405186407.wbieco004 (Accessed December 5, 2022).

[8] Bandura A Walters RH. Social learning and personality development. New York: Holt Rinehart and Winston (1963) (Social learning and personality development).

[9] Venkat M Janakiram C. Mass media coverage in health & oral health-related advertisements: a content analysis in Kerala, India. J Oral Biol Craniofacial Res. (2021) 11(3):451–6. 10.1016/j.jobcr.2021.06.001 PMC8253971 34258183

[10] Sandberg H Gidlöf K Holmberg N. Children's exposure to and perceptions of online advertising. Int J Commun. (2010) 5(0):30. ISSN: 1932-8036/20110021

[11] Charry KM. Product placement and the promotion of healthy food to pre-adolescents. Int J Advert. (2014) 33(3):599–616. 10.2501/IJA-33-3-599-616

[12] Greenberg BS Rosaen SF Worrell TR Salmon CT Volkman JE. A portrait of food and drink in commercial TV series. Health Commun. (2009) 24(4):295–303. 10.1080/10410230902889233 19499423

[13] Carden L Wood W. Habit formation and change. Curr Opin Behav Sci. (2018) 20:117–22. 10.1016/j.cobeha.2017.12.009

[14] Loewenstein G Price J Volpp K. Habit formation in children: evidence from incentives for healthy eating. J Health Econ. (2016) 45:47–54. 10.1016/j.jhealeco.2015.11.004 26717440

[15] Gardner B Rebar AL. Habit formation and behavior change. Oxford research encyclopedia of psychology (2019). Available at: https://oxfordre.com/psychology/view/10.1093/acrefore/9780190236557.001.0001/acrefore-9780190236557-e-129 (Accessed December 5, 2022).

[16] Harriger JA Serier KN Luedke M Robertson S Bojorquez A. Appearance-related themes in children's animated movies released between 2004 and 2016: a content analysis. Body Image. (2018) 26:78–82. 10.1016/j.bodyim.2018.06.004 29957304

[17] Marcenes W Kassebaum NJ Bernabé E Flaxman A Naghavi M Lopez A Global burden of oral conditions in 1990-2010: a systematic analysis. J Dent Res. (2013) 92(7):592–7. 10.1177/0022034513490168 23720570 PMC4484374

[18] Abanto J Carvalho TS Mendes FM Wanderley MT Bönecker M Raggio DP. Impact of oral diseases and disorders on oral health-related quality of life of preschool children. Community Dent Oral Epidemiol. (2011) 39(2):105–14. 10.1111/j.1600-0528.2010.00580.x 21029148

[19] Bönecker M Abanto J Tello G Oliveira LB. Impact of dental caries on preschool children's quality of life: an update. Braz Oral Res. (2012) 26(spe1):103–7. 10.1590/S1806-83242012000700015 23318751

[20] Levine RS. Childhood caries and hospital admissions in England: a reflection on preventive strategies. Br Dent J. (2021) 230(9):611–6. 10.1038/s41415-021-2945-8 33990749 PMC8120769

[21] Surgery F of D. The state of children's oral health in England (2015).

[22] Goodwin M Sanders C Davies G Walsh T Pretty IA. Issues arising following a referral and subsequent wait for extraction under general anaesthetic: impact on children. BMC Oral Health. (2015) 15(1):3–3. 10.1186/1472-6831-15-3 25595299 PMC4324052

[23] Selwitz RH Ismail AI Pitts NB. Dental caries. Lancet. (2007):369(9555):51–9. 10.1038/nrdp.2017.30 17208642

[24] Goodwin M Patel DK Vyas A Khan AJ McGrady MG Boothman N Sugar before bed: a simple dietary risk factor for caries experience. Community Dent Health. (2017) 34(1):8–13. 10.1922/CDH_3926Goodwin06 28561551

[25] Moynihan PJ Kelly SAM. Effect on caries of restricting sugars intake: systematic review to inform WHO guidelines. J Dent Res. (2014) 93(1):8–18. 10.1177/0022034513508954 24323509 PMC3872848

[26] England PH. Delivering better oral health: an evidence-based toolkit for prevention. 10.12968/denu.2008.35.7.460 18853715

[27] Peer models and children’s behavioral change—Dale H. Schunk (1987). Available at: https://journals.sagepub.com/doi/abs/10.3102/00346543057002149 (Accessed December 5, 2022).

[28] Young children’s recall and reconstruction of audio and audiovisual narratives on JSTOR. Available at: https://www.jstor.org/stable/1130375 (Accessed December 5, 2022). 10.1111/j.1467-8624.1986.tb00262.x 3757597

[29] Wrieden WL Levy LB. “Change4life smart swaps”: quasi-experimental evaluation of a natural experiment. Public Health Nutr. (2016) 19(13):2388–92. 10.1017/S1368980016000513 27002189 PMC4981896

