# Supplementary material for: Portrayal of oral hygiene and risk behaviours in animated movies
Source: Front Oral Health. 2023 Jul 5;4:1116717. doi: 10.3389/froh.2023.1116717 (PMC10354335; doi:10.3389/froh.2023.1116717)
Supplement: Supplementary file 1 [file Datasheet1.docx]

# **Supplementary Material**

1. **Overview of coding instrument used in the study**

**Coding instrument**

**a) Introduction and general instructions**

**Unit of analysis**

An exposure (scene) where an oral hygiene practice and/or risk behaviour is displayed or mentioned (or both) by one (or more) of the characters. Related behaviours include:

- Oral hygiene practices
  - brushing teeth,
  - flossing teeth,
  - using mouthwash,
  - rinsing
- Risk behaviours:
  - sugar consumption (both solid and liquid (i.e., drinks containing sugar), this can be both as consumed or simply preparing something to eat/drink with sugar (e.g., placing sugar cubes on a drink before drinking it)
  - smoking (use of tobacco more generally)
  - alcohol (drink containing alcohol)

**IMPORTANT**: only behaviours that are actively present in a scene will be coded. Actively present means a verbal presentation (someone’s talking about a behaviour), visual presentation (someone’s acting a behaviour) or a combination of both (someone’s acting a behaviour while talking about it). If background characters undertake a behaviour, this will not be coded.

**Scene**

A scene or exposure occurs when an oral hygiene practice or risk behaviour is displayed (or discussed) during the movie. If two or more characters are performing/mentioning the behaviour, these counts are 2 or more exposures.

**b) How to code scenes/exposures**

When a scene with an oral hygiene practice and/or risk behaviour appears, then PAUSE the movie and code the necessary information.

| Film ID |
| --- |
| Date of coding |
| Coder |
| Character name(s) |
| Nature of behaviour   - Oral hygiene practice(s)   - Brushing teeth   - Flossing teeth   - Using mouthwash   - Rinsing      - Risk behaviours   - Sugar consumption     - Food     - Drink     - Both   - Smoking     - Smoking     - Using tobacco     - Other, explain   - Alcohol |
| Mode of presentation   - Verbal - Visual - Combination |
| Valence of behaviour   - Positive - Negative - Neutral |
| Nature of character   - Good - Evil |
